# Pharmaceutical induction of ApoE secretion by multipotent mesenchymal stromal cells (MSCs)

**DOI:** 10.1186/1472-6750-8-75

**Published:** 2008-09-29

**Authors:** Suzanne Zeitouni, Brian S Ford, Sean M Harris, Mandolin J Whitney, Carl A Gregory, Darwin J Prockop

**Affiliations:** 1 Center for Gene Therapy, Tulane University Medical School, New Orleans, LA,70115, USA; 2 Institute for Regenerative Medicine, Texas A&M Health Science Center, Temple, TX,76502, USA

## Abstract

**Background:**

Apolipoprotein E (ApoE) is a molecular scavenger in the blood and brain. Aberrant function of the molecule causes formation of protein and lipid deposits or "plaques" that characterize Alzheimer's disease (AD) and atherosclerosis. There are three human isoforms of ApoE designated ε2, ε3, and ε4. Each isoform differentially affects the structure and function of the protein and thus the development of disease. Homozygosity for ApoE ε4 is associated with atherosclerosis and Alzheimer's disease whereas ApoE ε2 and ε3 tend to be protective. Furthermore, the ε2 form may cause forms of hyperlipoproteinemia. Therefore, introduction of ApoE ε3 may be beneficial to patients that are susceptible to or suffering from these diseases. Mesenchymal stem cells or multipotent mesenchymal stromal cells (MSCs) are adult progenitor cells found in numerous tissues. They are easily expanded in culture and engraft into host tissues when administered appropriately. Furthermore, MSCs are immunosuppressive and have been reported to engraft as allogeneic transplants. In our previous study, mouse MSCs (mMSCs) were implanted into the brains of ApoE null mice, resulting in production of small amounts of ApoE in the brain and attenuation of cognitive deficits. Therefore human MSCs (hMSCs) are a promising vector for the administration of ApoE ε3 in humans.

**Results:**

Unlike mMSCs, hMSCs were found not to express ApoE in culture; therefore a molecular screen was performed for compounds that induce expression. PPARγ agonists, neural stem cell conditioned medium, osteo-inductive media, dexamethasone, and adipo-inductive media (AIM) were tested. Of the conditions tested, only AIM or dexamethasone induced sustained secretion of ApoE in MSCs and the duration of secretion was only limited by the length of time MSCs could be sustained in culture. Upon withdrawal of the inductive stimuli, the ApoE secretion persisted for a further 14 days.

**Conclusion:**

The data demonstrated that pre-treatment and perhaps co-administration of MSCs homozygous for ApoE ε3 and dexamethasone may represent a novel therapy for severe instances of AD, atherosclerosis and other ApoE-related diseases.

## Background

Apolipoprotein E is a 34 kDa secreted lipoprotein, first discovered by Shore and Shore in 1973, see reference 1. Like other apolipoproteins, its primary function is to scavenge cholesterol, associated lipids and proteins for transport to the liver for processing. In healthy individuals, a plasma concentration of approximately 5 mg/dL is sufficient to maintain systemic lipid homeostasis [2]. Although primarily expressed in liver [1], ApoE contrasts with the other apolipoproteins in that it is also expressed in the spleen, lungs, kidneys, myoblasts, and macrophages [2,3]. ApoE is also highly expressed in the brain, by microglia and astrocytes [3,4] and there have also been reports of ApoE production by neurons [3]. Although poorly understood, the role of ApoE in the central nervous system is thought to be comparable to its role in plasma, sequestering cholesterol, lipids and other macromolecular debris from neural tissue [5].

The effectiveness of ApoE as a lipid scavenger is closely related to the ApoE isoform. ApoE polymorphism is thought to be uniquely exhibited by humans [3] in that it consists of three isoforms, ApoE ε2, ε3, and ε4. Although the isoforms vary only at two amino acid residues, the changes are sufficient to profoundly alter the tertiary structure and affect lipid binding properties of the protein [3,5]. ApoE polymorphism occurs at residues 112 and 158, ApoEε2 has two cysteines, ApoEε3 had a cysteine and an arginine and ApoEε4 has two arginines at these positions. ApoE ε3 is the most common isoform with an incidence of approximately 77% in the population [1,3,4], followed by ε4 in approximately 15%, and then ε2 at approximately 7%.

Due to the profound effects on the functional and structural properties of the protein, different isoforms of ApoE are associated with different diseases such as stroke, multiple sclerosis, Parkinson's disease, alcoholic cirrhosis of the liver and type 2 diabetes [3,6-8]. The role that ApoE isoforms play in disease development seems to be closely associated with different affinities of the various isoforms for the low density lipoprotein-receptor and lipids themselves. For instance in the case of Alzheimer's disease (AD), individuals that are homozygous or heterozygous for ApoE ε4, have a significantly increased risk of developing the disease with an earlier onset [3] compared with individuals with the most common ApoE ε3/ε3 genotype [1,3,4,9-11]. Likewise, individuals homozygous for Apo ε2 or with an ApoE ε2/ε3 genotype also have normal susceptibility for AD. Although ApoE4 is not an absolute determinant of the disease 40–65% of AD patients have at least one copy of the 4 allele. However, the role of ApoE isoforms is complex with additional reports also implicating ApoE ε2 with hyperlipidaemia and ApoE ε4 with hypercholesterolaemia. [9,12]. In consideration of the putative association of ApoE ε2 and ε4 with disease it is reasonable to assume that safe administration of ApoE ε3 could provide beneficial effects for some individuals.

Multipotent mesenchymal stromal cells (MSC), also known as marrow stromal cells or mesenchymal stem cells, are a heterogeneous population of non-hematopoietic cells representing 0.01–0.001% of the total bone marrow [13]. The *in vivo *function of MSCs remains controversial, but they are generally regarded as hematopoietic support cells and also a source of progenitors for structural tissues [14].

The existence of such cells was first suggested by Cohneheim in the 1870s [14] but Friedenstein and his colleagues were the first to isolate and culture MSCs in the 1970s [15-19], followed by Caplan and colleagues [20,21] who were the first to propose the phrase "mesenchymal stem cells". These cells are adherent to plastic and are known to differentiate to osteoblasts, chondrocytes and adipocytes *in vitro *and *in vivo *[13,14,22,23]. However, more recent findings suggest that MSCs may differentiate to additional cell types [23]. Another remarkable characteristic of MSCs are their immunosuppressive qualities, suggesting that they may survive and engraft in allogeneic recipients [13]. Because MSCs are easily expanded in culture, readily engraft into host tissues, and may locally modulate the immune response, they represent ideal candidates for cytotherapy. Specifically, MSCs represent an extremely useful tool for the treatment of deficits in ApoE function if harbouring the ApoE ε3 isoform.

In a previous study we demonstrated that murine MSCs (mMSCs) injected into the lateral ventricles of 4 day old ApoE null mice secreted small amounts of ApoE into the tissue [24]. As ApoE null mice mature, the animals develop cognitive impairment, but upon behavioural analysis, the presence of mMSCs resulted in improved cognitive behavioural testing compared to the control groups [24]. Therefore, human MSCs (hMSCs) seemed a promising vector for the administration of ApoE ε3 in human diseases. In contrast to mMSCs, unmanipulated human MSCs (hMSCs) do not synthesize ApoE mRNA *in vitro*, but expression could be observed after 7 days of adipocyte differentiation [25]. Pharmaceutical induction of ApoE secretion has been demonstrated in macrophages, hepatocytes, adipocytes and other cell types by a variety of agents. In two studies employing rat hepatocytes, ApoE secretion was stimulated using dexamathasone, insulin, or a combination of both [26,27]. In macrophages, secretion of ApoE was induced by treatment with TGFβ and dexamethasone [28]. Expression of ApoE mRNA by adipocytes was increased by pioglitazone and ciglitazone, neither of which affected the ApoE secretion of macrophages [29].

This study examined pharmaceutical induction of endogenous ApoE expression by hMSCs. We demonstrate here that dexamethasone alone or adipo-inductive media (AIM) containing dexamethasone, indomethacin and isobutylmethylxanthine resulted in the expression of high levels of ApoE by hMSCs *in vitro*. Maximal expression could be attained after approximately 5–10 days of dexamethasone or AIM treatment depending on the donor source of the cells. We found no correlation between the ApoE expression levels and the degree of cell expansion prior to the assay, nor did we find any correlation between sex or age and ApoE secretion kinetics. The maximal rate of secretion ranged between 0.004–0.006 ng cell^-1 ^day^-1^. After withdrawal of the stimulus, ApoE expression remained approximately 14 days, but sometimes much longer, depending on the donor. These *in vitro *results demonstrate that ApoE expression by hMSCs is entirely possible through pharmaceutical induction without the necessity for genetic manipulation. Therefore, MSCs may represent a safe and feasible strategy for treatment of diseases that result from a functional deficit of ApoE.

## Results

### Culture and characterization of human MSCs

**Figure 1:**
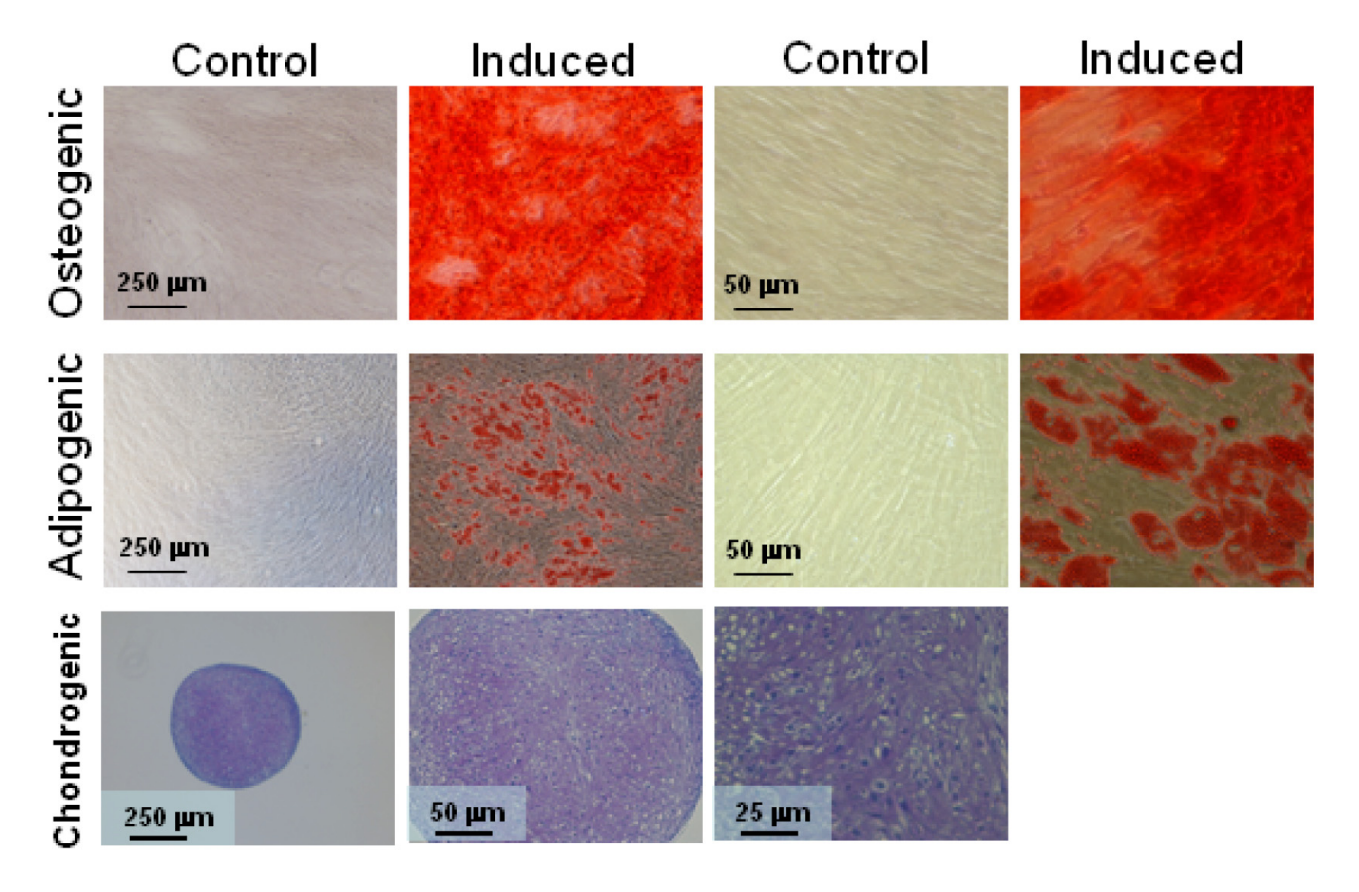
**Differentiation of human MSCs**. Characterization of MSCs by differentiation assays. One donor is presented. Row 1, high (right) and low power (left) micrographs of osteogenic MSCs stained with Alizarin Red S for calcium. Row 2, high (right) and low power (left) micrographs of adipogenic MSCs stained with oil red O for lipid. Row 3, high (right) and low power (center and left) micrographs of toluidine blue (purple) stained sections of MSC pellets induced to form chondrocytes and cartilage. All donors were assayed for adipogenesis and osteogenesis with the exception of chondrogenesis, where 2 donors were assayed.

We employed MSCs from a total of eight donors for the study. Human MSCs were recovered from the mononuclear fraction of whole bone marrow and cultured as described in the *Materials and Methods*. The adherent component of the cultures, containing hMSCs is relatively pure, but frequently contains traces of contaminating osteoblasts, fibroblasts and senescent cells. To assay for enrichment of multipotent hMSCs, we performed differentiation assays to osteoblasts, adipocytes and chondrocytes. The hMSCs readily differentiated into all three lineages when subjected to the appropriate conditions (Figure 1). MSCs formed mineralized osteoblasts as detected by the calcium binding dye, alizarin red S; they formed adipocytes with oil red O stainable lipid droplets; and formed proteoglycan-filled cartilage pellets that stained purple with toluidine blue when subjected to pellet culture in the presence of bone morphogenic protein 2 and tumor necrosis factor β.

### Induction of ApoE production

**Figure 2:**
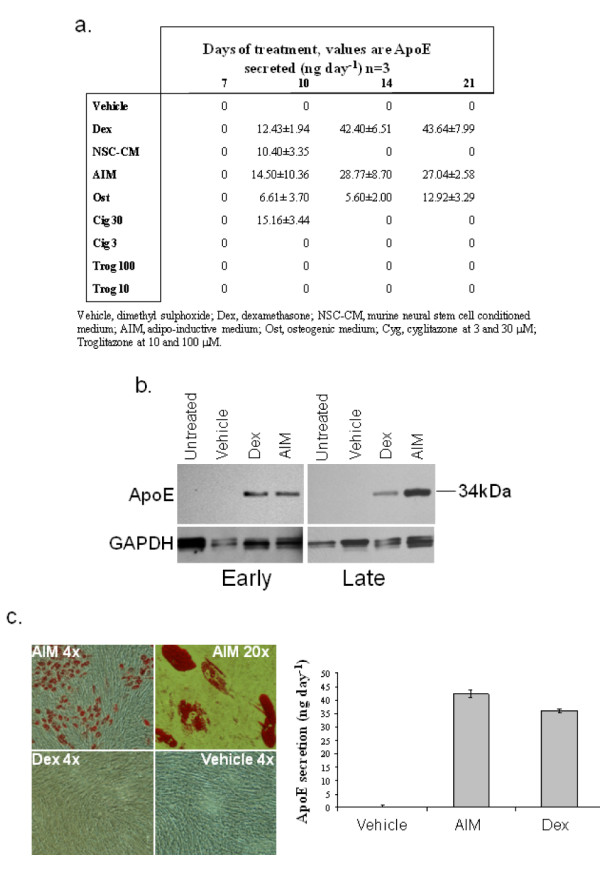
**a – Assays of ApoE production**. Some conditions known to induce ApoE production in other cells and their effect upon MSCs. NSC conditioned media AIM and osteogenic conditions are also included. Experiment was repeated with 2 donors and data from one of the donors is presented, n = 3, average ± standard deviation is shown. b – Detection of ApoE in MSCs by western blotting. Western blot demonstrating ApoE (34 kD) production (upper) in early (passage 2, left column) and late (passage 8, right column) passage MSCs. Lanes were normalized to GAPDH expression (lower). Experiment was repeated 5 donors. c – Dex-mediated expression of ApoE in MSCs is not accompanied by adipocyte differentiation. ApoE expression by MSCs was induced by exposure to Dex or AIM. After 21 days, the monolayers were stained with oil red O to visualize lipid droplets (left). Lipid droplets were evident in AIM treated, but not Dex treated MSCs. However, ApoE expression was high in both cases when media was assayed by ELISA (right).

Unmanipulated hMSCs did not secrete detectable levels of ApoE when the conditioned culture media was assayed by ELISA as shown in the controls in figures 2, 4, 5 and 6. Since previous studies have reported successful induction of ApoE secretion from other cell types, we decided to screen some candidates for ApoE inducing activity. A total of nine conditions were tested; 0.5 μM dexamethasone (Dex), neural stem cell conditioned media (NSC-CM), AIM, osteogenic media (ost), 30 μM ciglitazone (cig 30), 3 μM ciglitazone (cig 3), 100 μM troglitazone (trog 100), 10 μM troglitazone (trog 10). Confluent MSC monolayers from 2 donors were cultured in the experimental conditions for 21 days. At 2 day increments, conditioned medium was recovered for ApoE ELISA. Of the conditions tested, five induced ApoE expression after 10 days of treatment; these were Dex, NSC-CM, AIM, ost and cig 30 (Figure 2a). Cig 30 transiently induced ApoE secretion by day 10 of culture but these levels were absent at subsequent time points. Ost maintained ApoE expression to day 21, the end point of the experiment, but the levels remained very low. Since mMSCs were shown to secrete ApoE in brains of mice [24], we tested conditioned media from murine neural stem cells. Transient expression of ApoE was induced after 10 days of culture, but these levels were undetectable at later time points. Since the ELISA detects only human ApoE, the levels were derived from the hMSCs rather than trace levels in the conditioned media itself. In contrast, Dex and AIM induced high and robust expression of ApoE, which persisted until the end of the experiment at 21 days.

**Figure 3:**
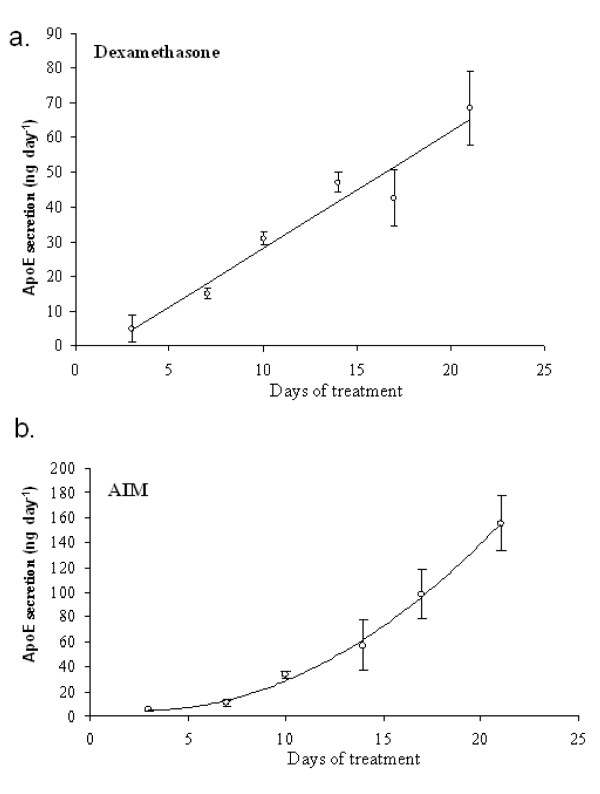
**ApoE production kinetics**. ApoE production during 21 days of treatment with 0.5 μM dexamethasone (panel a) or adipoinductive media (panel b). Experiment was repeated for 8 donors, data from one donor is presented, n = 3, error bars are standard deviations.

**Figure 4:**
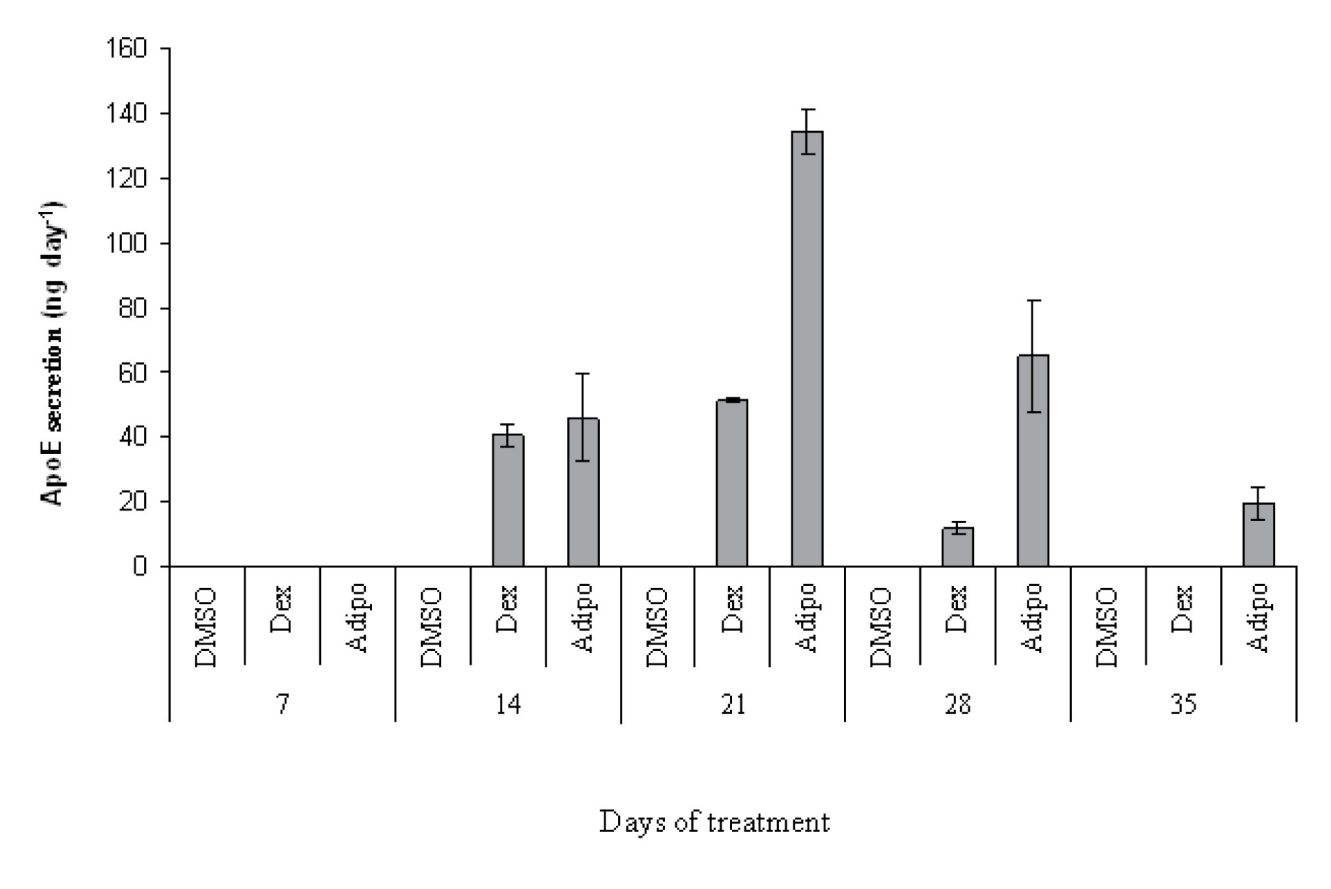
**ApoE production following withdrawal of inductive media**. MSCs were treated with the inductive media (0.5 μM dexamethasone (Dex) or AIM (Adipo)) through for 21 days, then with CCM for a further 2 weeks. ApoE levels drop abruptly after day 21. Experiment was repeated 3 donors, data from one donor is presented, n = 3, error bars are standard deviations.

**Figure 5:**
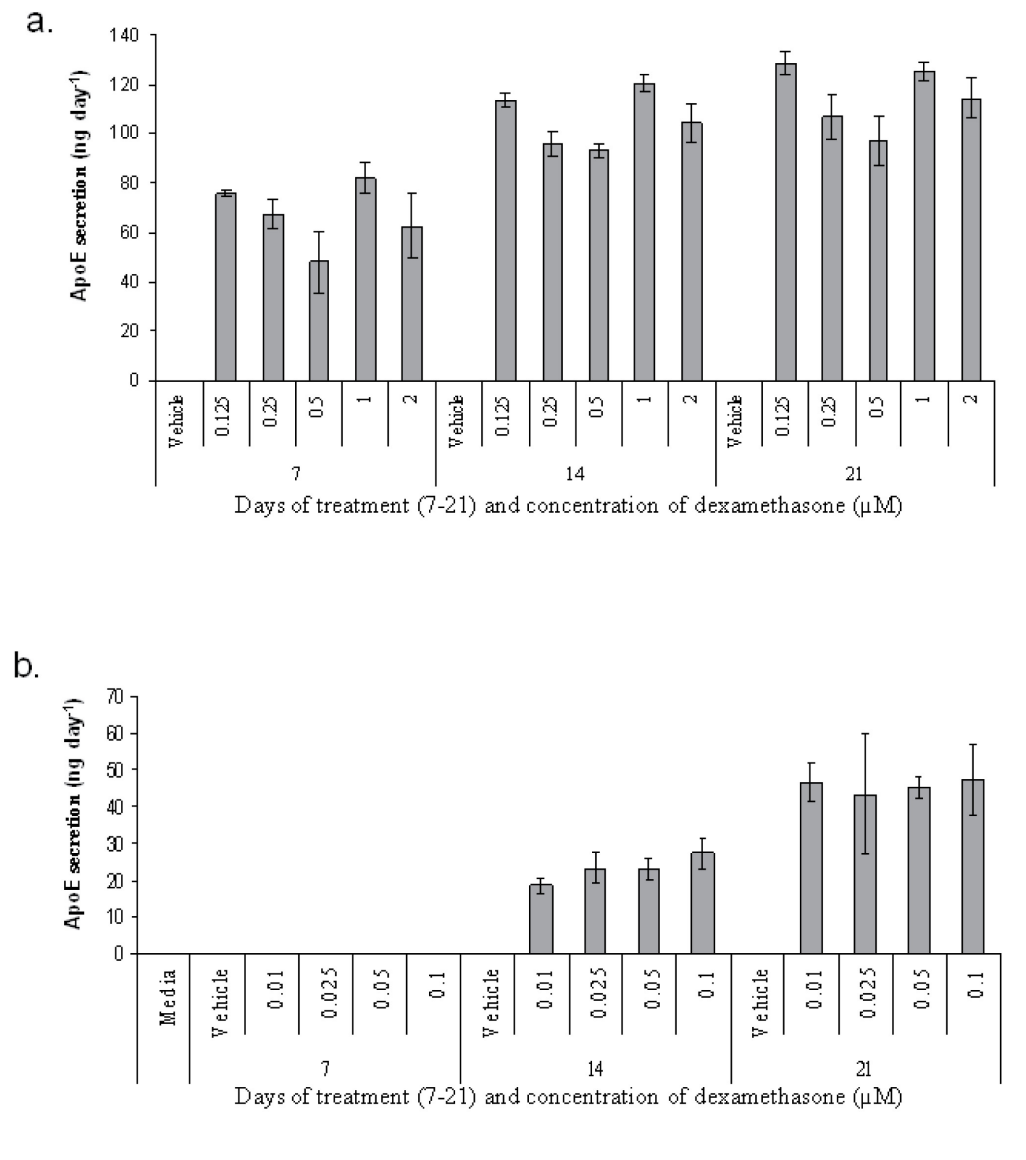
**Dose responses**. Dexamethasone dose response (high dose, panel a, and low dose, panel b). Experiments were repeated with 2 donors, data from one of the donors is presented, n = 3, error bars are standard deviations.

**Figure 6:**
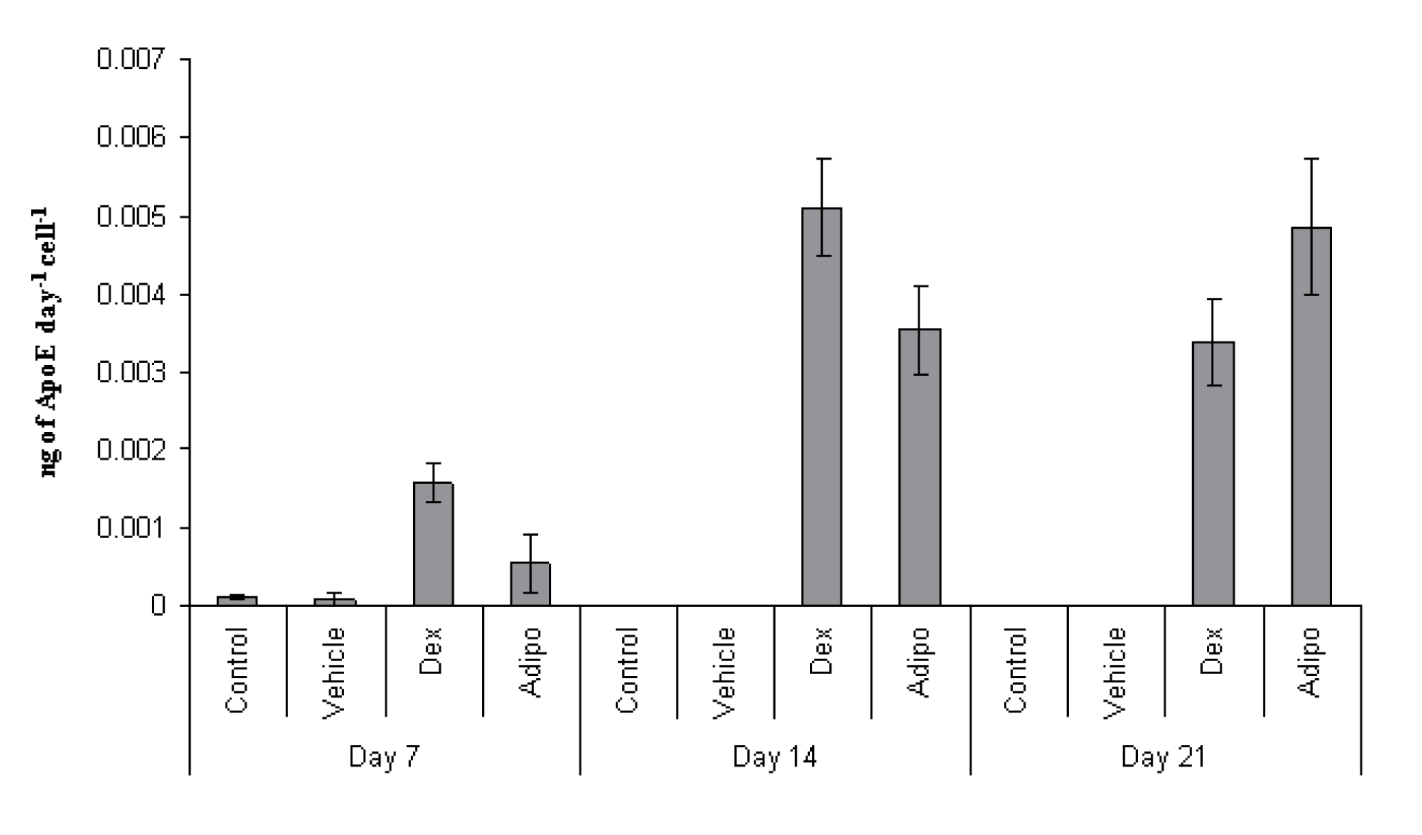
**ApoE production per cell**. Rate of ApoE production normalized to cell number. Cells were treated with inductive media (0.5 μM dexamethasone (Dex) or AIM (Adipo)) for 7–21 days. Experiment was performed on 1 donor, n = 3, error bars are standard deviations.

To confirm ELISA data, ApoE was also detected in protein extracts from MSCs treated with Dex, AIM and control media, containing an appropriate level of vehicle DMSO, by western blotting. A single band (34 kDa) corresponding to ApoE was present only in the Dex and AIM treated MSCs (Figure 2b). The position of this band was later confirmed by comparison to immunoblotted recombinant human ApoE (data not shown). To confirm that Dex was solely responsible for the induction of ApoE secretion, we tested whether the two additional components of AIM induced ApoE expression. Isobutyl-methyl xanthine, a phosphodiesterase inhibitor, and indomethacin, a ligand of the adipogenesis master-regulator peroxisome proliferator-activated receptor, both failed to induce ApoE expression alone or in combination, nor did they induce terminal adipogenesis (data not shown). Furthermore, Dex did not appear to induce ApoE as a by-product of terminal adipogenesis, since lipid droplets could not be detected in long term treated cultures (Figure 2c). Thereafter, Dex and AIM were the selected conditions for subsequent experiments.

### Kinetics and Maintenance of ApoE production

To examine the kinetics, longevity, and donor-dependency of the inducible ApoE secretion, a series of time course experiments were performed with hMSCs from eight donors. In the first series of experiments, we examined the rate of response to Dex and AIM in terms of attaining maximal ApoE secretion. Confluent monolayers of hMSCs were established from passage 2 (approximately 15 doublings per cell) cultures. Cells were treated with Dex or AIM and for controls, the cells were treated with either vehicle (DMSO) or not at all. At 2–3 day intervals, media were recovered and ApoE levels were measured by ELISA (Figure 3). For all MSC preparations tested the control cultures did not secrete ApoE over the entire experimental period (data not shown). For the AIM and Dex conditions, maximal ApoE expression was attained after 21 days and was sustained for up to 35 days. After long term culture (about 35 days), the ApoE yield dropped due to apoptosis of the MSCs that frequently occurs after sustained culture at confluency. The ApoE yields were comparable between all of the MSC preparations at about 70–100 ng day^-1 ^for the Dex (Figure 3a) and 100–140 ng day^-1 ^for the AIM conditions (Figure 3b). There were no correlations between the rate of response to stimulus and donor age or sex. When stimulated by Dex only, there was a linear increase in the rate of ApoE secretion from hMSCs. However, there was an exponential increase in the rate of ApoE secretion when treated with AIM and this was accompanied by differentiation into adipocytes. Since adipocytes are a source of ApoE *in vivo*, this probably accounts for the higher overall ApoE output, and the accelerated rate of secretion.

The next study was designed to address the longevity of ApoE secretion after withdrawal of stimulus. Confluent passage 2 MSC cultures were incubated in Dex and AIM media for 21 days to attain maximal expression of ApoE. The cells were then cultured in the absence of stimulus for an additional 2 weeks (Figure 4). After 2 weeks, ApoE expression dropped to about 8% of the maximum for AIM treated cells and the Dex treated cells expressed barely detectable levels. This suggested that for sustained ApoE expression, induction is required at least weekly.

In consideration of the prospect of clinical application, we examined the dose dependency of ApoE induction on MSCs. Although AIM media was most effective in inducing ApoE secretion by MSCs, the compounds have not been safely co-administered *in vivo*. On the other hand, dexamethasone is a standard therapeutic and would be acceptable for administration to humans at low doses. We performed dose response curves ranging from 0.125 μM to 2 μM and 0.01 μM to 0.1 μM found that the dose of Dex could be reduced from 0.5 μM to 0.125 μM with insignificant reduction in ApoE secretion (Figure 5a). The lower dose response series demonstrated that approximately half maximal ApoE expression could be attained (Figure 5b).

### ApoE production and cell number

To provide insights on cell viability and proliferative potential during induction, we examined ApoE secretion on a per cell basis. MSCs were allowed to reach 70% confluency before beginning treatment with AIM or Dex. Media and cells were collected at day 7, 14 and 21. At each time point, the amount of ApoE secreted per day was normalized to the number of cells in the monolayer. Overall cell recovery was slightly reduced when compared to the untreated controls suggesting that DMSO primarily affected MSC proliferation (data not shown). However, the presence of Dex and AIM did not affect long term viability, since the cultures slowly expanded over the 21 day experimental period. In the case of AIM, normalized ApoE levels reflected the unmodified measurements (Figure 3b) with a continuous increase in ApoE levels over time suggesting that as the cells divide in culture, they retain their ApoE secretion potential. In the case of the Dex conditions, normalized ApoE levels dropped slightly, suggesting expansion of some cells with limited or no potential for ApoE secretion (Figure 6).

**Figure 7:**
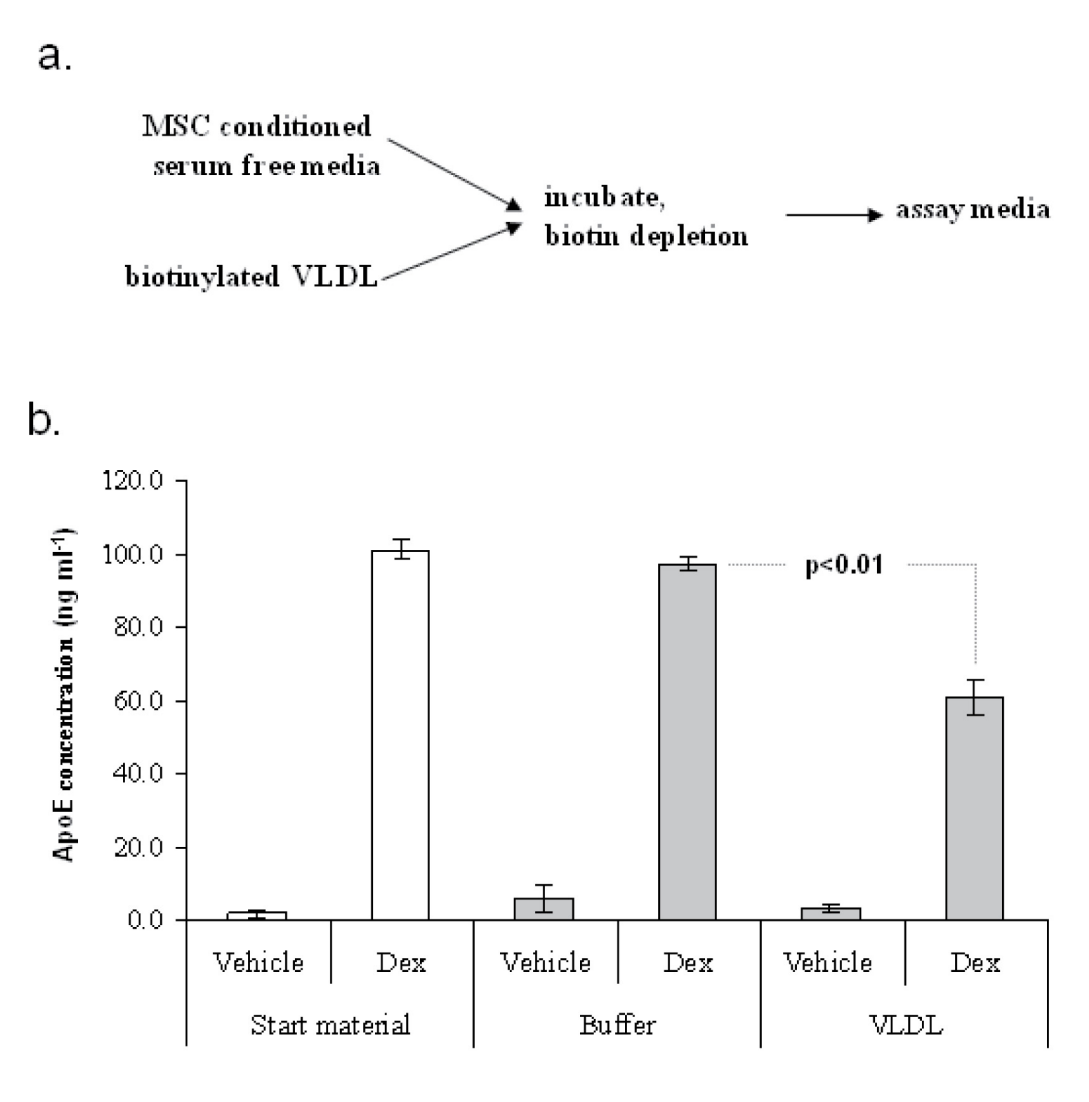
**Binding of ApoE to VLDL**. ApoE was secreted into serum free media by MSCs. The conditioned media was then added to biotinylated VLDLs (panel a). After biotin depletion, the remaining ApoE in the conditioned medium was measured by ELISA (panel b). When compared with buffer alone, biotinylated VLDLs depleted the ApoE from the conditioned medium. Data from one of two donors is presented, n = 3, error bars are standard deviations.

**Figure 8:**
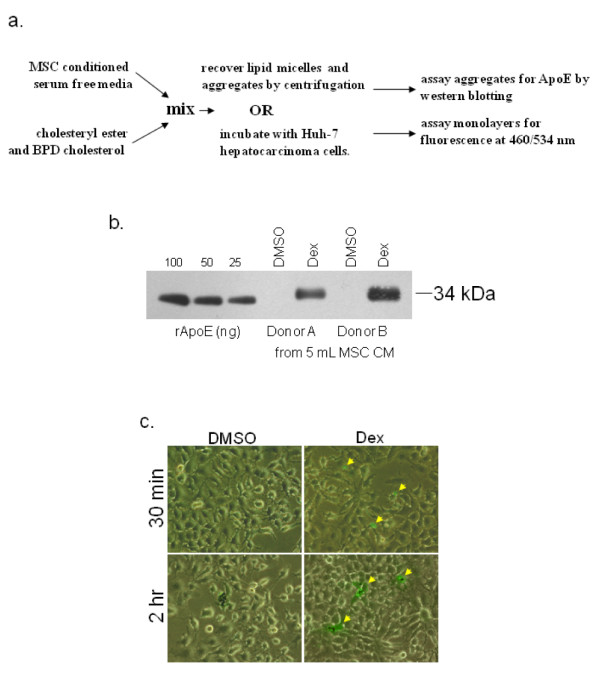
**Binding of ApoE to synthetic micelles and uptake by hepatocyte cells**. ApoE was secreted into serum free media by MSCs. Fluorescent cholesterol-ester and cholesterol was then added to the media (panel a). Upon recovery of the micelles by centrifugation, ApoE could be detected when analyzed by western blotting (panel b). When ApoE conditioned media was mixed with lipids and incubated with huh-7 hepatocarcinoma cells, it accelerated uptake when compared with control media. Phase contrast/fluorescent merge indicating hepatocytes taking up fluorescent cholesterol (green) from the medium (panel c).

We then tested the ability of the ApoE to bind to VLDLs. To reduce background, ApoE expression was induced for 2 days by Dex treatment in serum free media. MSC viability was not significantly affected during the brief exposure to serum free conditions and ApoE expression occurred at levels comparable to expression in serum containing media (Figure 7b). The conditioned media was then incubated with biotinylated VLDLs and then subjected to a series of biotin-mediated depletions using streptavidin coated microtiter plates (Figure 7a). Upon ELISA of the depleted media, ApoE levels were significantly reduced when compared to controls that were treated identically, but lacked VLDL (Figure 7b). This suggests that the MSC-derived ApoE meets at least one of its *in vivo *functions. ApoE also binds to the LDL receptor and facilitates internalization by hepatocytes, macrophages and astrocytes. To examine the potential of MSC-derived ApoE to accelerate lipid uptake, we generated synthetic micelles containing fluorescently labelled cholesterol ester and free cholesterol. When such lipid micelles were added to serum free media conditioned by the ApoE (Figure 8a), it bound to them and could be enriched from the media by centrifugation (Figure 8b). When compared to untreated controls, the ApoE conditioned media catalysed the formation of larger micelle aggregates, many of which could be identified by the naked eye (data not shown). Furthermore, ApoE conditioned media accelerated the uptake of the fluorescent lipid aggregates by huh-7 hepatocytes (Figure 8c).

## Discussion

There are a number of reports where ApoE has been successfully administered via direct gene delivery or virally in a variety of disease models including Alzheimer's disease [11], hypercholesterolemia [33-36], hyperlipidemia [37], atherosclerosis [37-39], experimental stroke [40] and hematopoietic diseases [41]. Direct gene introduction or direct viral therapy can be efficient and sustainable in some cases, but it is associated with safety concerns. To circumvent some of these problems, ApoE has been administered through hematopoietic stem cells (HSCs) in rodent models [42-44]. The HSCs are administered to radioablated animal models and long term hematopoietic stem cells reconstitute the bone marrow compartment. In some studies, ApoE is introduced to the HSCs virally, and in others, the inherent ApoE is utilized mostly through expression by resultant macrophages. These studies suggest that bone marrow transplantation is a promising vector for cytotherapeutic ApoE administration but the process of radioablation in humans is a dangerous procedure, and graft versus host disease is a common problem with allogeneic recipients. Viral integration, especially with long term repopulating HSCs is also a source of concern for these strategies.

In consideration of all of these caveats, a safer cytotherapeutic vector would be beneficial. MSCs are safely recovered from humans via a simple iliac crest aspirate and can be expanded by the million [22,31]. Their characteristics suggest that they would be an excellent delivery tool for ApoE; for instance, when administered into animal models, they survive for long periods in the brain (up to 6 months) and subcutaneously [24,45-48]. When in the brain, hMSCs secrete trophic factors that stimulate neural stem cell proliferation and probably their own survival [49]. Upon subcutaneous implantation, the hMSCs distribute themselves close to capillary beds suggesting that they have the capacity to secrete proteins into the blood [45]. MSCs are also immunomodulatory, with the capacity to suppress a variety of rejection mechanisms [11], reducing the possibility of graft versus host complications, and improving the probability of allogeneic transplant. Finally, transplanted hMSCs do not rapidly proliferate in adult tissues in the same way repopulating HSCs do, suggesting that they may also be safer for accommodating viral transgenes.

In our previous study [24], mMSCs were administered to the lateral ventricles of ApoE null mice. As they age, untreated ApoE null mice develop cognitive deficits that resemble Alzheimer's disease in addition to profound atherosclerotic pathogenesis. Remarkably, upon administration of the murine MSCs, the mice exhibited signs of improvement in some of the behavioural tests. When the brains of the experimental animals were examined, a small amount of murine ApoE could be detected. Although the presence of other mMSC derived factors could have contributed to functional recovery, the presence of ApoE 6 months after MSC administration strongly suggested that the functional deficit of ApoE in the null mice had been attenuated. We therefore hypothesized that hMSCs may provide a promising vector for administration of the ApoE3 isoform for the treatment of Alzheimer's disease and possibly atherosclerosis.

We could not detect ApoE expression *in vitro*, when cultured under standard conditions, nor could we detect expression when cultured in murine neural conditioned medium. This was surprising, since administration of murine MSCs *in vivo*, stimulated expression. We hypothesize that the ApoE null environment may have been sufficient to stimulate expression or more likely, the hMSCs required contact with the neural cells to initiate expression. Due to the limited availability of human neural tissue, and in view of potential inter-species incompatibility, we decided to attempt induction of expression using commonly available pharmaceuticals. This approach has been utilized with a variety of cell lines and a variety of reagents [26-29] and also in MSCs when differentiated completely into adipocytes [25]. Administration of adipocytes directly into the brain cannot be performed, therefore we tested related conditions in an attempt to induce ApoE expression without lipid droplet accumulation that occurs during adipogenic differentiation. In contrast with previous literature where adipocytes were employed [29], PPARγ agonists gave very limited success (Figure 2a) demonstrating that the compounds function in a cell type-dependent manner. Another series of studies demonstrated efficacy with the hydrocortisone analog, dexamethasone on murine macrophages and rat hepatocytes [26-28]. We therefore tested dexamethasone at the equivalent concentration employed for adipogenic differentiation and we achieved high levels of ApoE expression without complete differentiation into adipocytes. Indomethacin and Dex also induced ApoE secretion, but we also observed high levels of unwanted adipogenesis (data not shown). We acquired the genotype of 2 of the 8 donors and found them to be Apo ε3/3 and Apo ε2/3. These donors had comparable ApoE expression levels when compared with the other uncharacterized MSC preparations. This confirmed that MSCs with preferable Apo ε3/3 genotype would be compatible with the Dex induction strategy.

Permanent induction of ApoE would be beneficial, dismissing the need for constant drug administration. However, withdrawal of both Dex and AIM caused a reduction of ApoE output over 2 weeks. Although it is unclear whether this is due to outgrowth of a non-expressing component of MSCs or due to attenuation of expression, the extent of proliferation required to completely ablate expression is not expected in these confluent cultures. Of interest, is the reduced expression of ApoE in the AIM treated cultures, which, by day 21 have mostly differentiated into lipid-filled adipocytes. It appears that ApoE is not a prerequisite for maintenance of the terminal adipocyte phenotype. We did not observe a qualitative reduction in lipid filled cells suggesting that dedifferentiation had not occurred, but this phenomenon cannot be completely discounted.

We found that doses as low as 0.125 μM were sufficient to maintain maximal expression of ApoE, and doses as low as 0.01 μM could sustain half maximal levels of ApoE expression. These levels are comparable with published therapeutic plasma levels of dexamethasone, which are in the region of 0.001 – 0.05 μM for immunomodulation [50]. These results suggest that relatively safe plasma levels that can be attained clinically would be sufficient for ApoE induction by implanted MSCs.

Nevertheless, chronic Dex administration is not without serious side effects [51] such as immunological inhibition, diabetes/hyperglycemia, osteoporotic symptoms, gastric irritation, weight loss or gain, glaucoma, muscle pain/weakness and exacerbation of psychosis. The side effects increase with duration and dose. Since expression of ApoE was maintained for at least 7 days by hMSCs after withdrawal of stimulus, it is possible that less frequent administration of Dex (every 2 days, for instance) may maintain ApoE levels while reducing the probability of harmful effects. Furthermore, corticosteroids with faster clearance and shorter effect durations, such as prednisone or hydrocortisone may improve the risk/benefit ratio [51].

In terms of efficacy, we found that maximal ApoE expression per cell was in the region of 0.0033 ng per cell in 24 hr when treated with Dex. Since the plasma concentration is about 50 mg L^-1 ^[2], and the average blood volume in an adult human is 5.5 L, it would take an implant containing 250 million cells about 7 days to attain 1% of normal systemic ApoE levels, a dose that is protective against atherosclerotic plaque formation [38]. However, systemically infused MSCs have the capacity to migrate to sites of injury and inflammation, suggesting that the local dose of ApoE might be much higher at the lesions [14,22,23]. Direct injection of a lower dose of MSCs into the brain may provide long term relief of Alzheimer's disease while stimulating the local population of neural stem cells to repair existing damage [49]. In healthy humans, the blood brain barrier (BBB) is somewhat resistant to Dex penetration due to multidrug resistance receptor action [52] and this is a potential problem for treatment of Alzheimer's disease. There are, however, alternative corticosteroids, such as prednisolone, which are predicted to have similar effects and have been reported to have increased permeability into the cerebrospinal fluid [51,53-55]. Furthermore, there is extensive evidence that the BBB is compromised in individuals with Alzheimer's disease suggesting that Dex may have access to implanted MSCs in severe cases [56-58].

It can be argued that MSCs may not require pharmaceutical ApoE induction at all since there have been reports that they express various neuronal markers *in vitro *[59] and *in vivo *[46,59,60] and thus may spontaneously differentiate into astrocytes in the brain. Indeed, murine MSCs were shown to express nestin, β-III tubulin, neurofilament marker and GFAP (an astrocyte marker) *in vitro *[59], and GFAP, β-III tubulin, and neurofilament *in vivo *after transplantation into mice [46,59]. Human MSCs were also shown to express GFAP [60]*in vivo*. However, it is noteworthy that in every instance where MSCs were implanted into the murine brain, only a small proportion expressed the astrocyte marker, GFAP. Therefore, although some hMSCs may differentiate into ApoE-secreting astrcocytes, it remains important to pre-treat the hMSCs with Dex to ensure that the majority of the cells secrete ApoE immediately upon implantation.

Since functional ApoE has been expressed in *E. coli *[61] and in insect cells [62], it is likely that the MSC-derived ApoE is also functional. Nevertheless, we confirmed that the protein could associate with cholesterol esters and could also bind to VLDLs (Figure 7 and 8). MSC-derived ApoE also accelerated uptake of lipid by hepatocytes (Figure 8). Since the physiological role of ApoE is dependent binding of lipid and the LDL-receptor, these data suggest that the ApoE would satisfy its role *in vivo*. Of particular note, is the observation that MSCs produce high levels of ApoE in the absence of serum (Figure 7a). This raises the possibility of generating ApoE preparations for therapeutic use without the necessity for MSC administration. Since MSC conditioned media inherently contains neuroprotective cytokines [49], dialysed conditioned media containing ApoE may represent an efficacious neuroprotective cocktail for clinical use.

## Conclusion

In this study we have shown that expression of potentially therapeutic levels of functional ApoE by hMSCs can be induced with dexamethasone. These data demonstrate that co-administration of hMSCs genotyped for homozygous expression of ApoE ε3 and chronic cortisol treatment may represent a novel therapy for severe instances of ApoE related diseases.

## Methods

### Culture of human multipotent stromal cells

Mutlipotent stromal cells were acquired from the Tulane University adult stem cell distribution facility. In accordance with institutional review board approved protocols, the cells were prepared from 2 mL posterior iliac crest bone marrow aspirates derived from 7 males and 1 female between 25 and 34 years of age. After a brief interval of monolayer culture to exclude non-adherent hematopoietic cells, hMSCs were expanded to 70% confluency prior to passage or use in experiments. Cells were cultured according to standard MSC culture conditions in complete culture medium (CCM), consisting of alpha minimal essential medium (GIBCO, Invitrogen, Carlsbad, CA) containing 20% (v/v) FBS (Hyclone, Logan, UT and Altanta Biologicals, Norcross, GA), 2 mM L-glutamine and 100 units ml^-1 ^penicillin and 100 μg ml^-1 ^streptomycin (GIBCO, Invitrogen) [30,31].

### Differentiation of human multipotent stromal cells

To confirm that the cell preparations from the mononuclear layer were multipotent, a panel of differentiation assays were performed.

#### Osteogenic differentiation and Alizarin Red S staining

For osteogenic differentiation, confluent monolayers of hMSCs were incubated in CCM supplemented with 10^-8 ^M dexamethasone, 50 μg mL^-1 ^ascorbic acid and 5 mM β-glycerol phosphate (Sigma, Poole, UK) for 21 days with changes of medium every 2 days. The monolayers were then stained with 40 mM Alizarin Red S pH 4.0 (Sigma) for 30 min and washed 4 times with distilled water. Micrographs were taken using an inverted microscope (Nikon Eclipse, TE200).

#### Adipogenic differentiation and Oil Red O staining

All reagents were purchased from Sigma. Confluent monolayers of hMSCs in 6 well plates (10 cm^2 ^per well) were incubated in adipoinductive medium (AIM) consisting of CCM containing 0.5 μM dexamethasone, 5 × 10^-8 ^M isobutylmethylxanthine, and 5 × 10^-7 ^M indomethacin (Sigma). Media was changed every 2–3 days. After 21 days, the adipogenic cultures are fixed in 10% formalin for 15 min and stained with fresh Oil Red-O solution 0.5 (w/v) (Sigma) in 30% (v/v) isopropanol in phosphate buffered saline (PBS) for 20 min. The dishes were washed 3 times with excess PBS and visualized using an inverted microscope (Nikon Eclipse, TE200).

#### Chondrogenic differentiation and processing

Micromass pellet chondrogenic differentiation was carried out in accordance with the protocol of Sekiya *et al. *on 200,000 pelleted cells [32]. After 21 days of differentiation, the chondroid pellets were washed in PBS and fixed in 4% paraformaldehyde. The pellets were then embedded in paraffin, sectioned, then stained with toluidine blue to visualize sulphated proteoglycans and chondrocyte lacunae [31].

### ELISA detection of ApoE

#### Sample preparation for ELISA

hMSCs were plated in each well of a six well plate at 1000 cells per cm^2 ^and grown to 70% confluency. The media was then changed to the appropriate conditions and maintained for the appropriate duration with changes every 2–3 days. All experiments were performed in triplicate. Media samples were taken at intervals defined in the *Results *and stored for ELISAs at -20°C. Dexamethasone and dimethyl sulphoxide (vehicle) were purchased from Sigma and diluted into CCM from a 1000× stock solution. AIM and osteogenic media were prepared as described above and the individual components were added to CCM from stocks. For production of NSC conditioned medium, a frozen vial of murine neural stem cells (NSCs, a gift from Dr Jeffrey Spees, Department of Medicine, Cardiovascular Research Institute, University of Vermont) was employed. NSC conditioned medium was produced by incubation of the NSCs in NSC media [neurobasal alpha medium containing B27 supplement, L-glutamine, penicillin, streptomycin (Invitrogen), 10 ng mL^-1 ^epidermal growth factor, 10 ng mL^-1^, fibroblast growth factor (Sigma)] for 2 days. NSC conditioned medium was mixed 1:1 with CCM prior to addition to the hMSCs. Ciglitazone was purchased from Tocris Biochemical (Bristol, UK) and troglitazone was purchased from Cayman Chemical (Ann Arbor, MI). The drugs were added to CCM from a stock dissolved in DMSO. All assays were conducted in parallel with vehicle, or unconditioned media controls.

#### Human ApoE sandwich ELISA

A polyclonal goat-anti-human ApoE antibody (Academy Biomedical, Houston, TX) was diluted in PBS at 1:1000. Each well of a high binding microtiter plate (Fisher Lifesciences) was coated with 100 μL of the antibody solution for 15 hrs at 4°C. The coating solution was then removed followed 3 × 5 min washes with 150 μL PBS. Wells were then blocked by addition of PBS containing 0.1% (v/v) Tween20 (Fisher Lifesciences) and 5% (w/v) bovine serum albumin (Sigma) for 2 hrs at room temperature. Block solution removed and the wells were washed for 3 × 5 min in PBS containing 0.1% (v/v) Tween20 (PBST). Media samples (100 μL) were added to the wells in triplicate. Standard solutions of recombinant human ApoE (Calbiochem, Gibbstown, NJ) were diluted in PBST. Samples were incubated in the microtiter plates for 15 hrs at 4°C. Plates were then washed for 3× for 5 mins in PBST, and the detection antibody (100 μL) was added consisting of a 1:2000 dilution of HRP conjugated goat anti human ApoE IgG (Academy Biomedical) in PBST at room temperature. After 2 hrs, the wells were washed in PBST and developed by addition of 100 μL TMB (Pierce, Rockford IL).

After approximately 5 mins, the reactions were stopped with the addition of 50 μL 2 N sulphuric acid (Fisher Lifesciences) and the resultant yellow substrate was measured by absorbance (450 nm) on a 96 well plate reader (Fluostar Optima, BMG).

#### Cell number evaluation

Cells were counted using a DNA intercalating dye that fluoresces upon incorporation (CyQuant dye, Invitrogen). Briefly, monolayers were recovered by trypsinization, washed in PBS, then resuspended in lysis buffer (PBS containing 0.1% Triton ×100, 1 mM MgCl_2_) containing a 400 fold dilution of the CyQuant dye. At various dilutions, the labelling suspensions were aliquoted into a microtiter plate and fluorescence was measured at 480/525 nm on a 96 well plate reader (Fluostar Optima) using opaque black plates (Fisher Scientific). Fluorescence was proportional to DNA content, and thus cell number, when compared to known cell standards.

### ApoE detection by western blotting

#### Sample preparation

MSCs were plated on 15 cm plates at 100 cells per cm^2 ^and grown to 70% confluency. The cells were then maintained under experimental conditions for 21 days and recovered by scraping. The media was collected for ELISAs. Cells were washed in PBS, flash frozen in liquid nitrogen and then stored at -80°C for protein extraction. The proteins were solubilized using an extraction solution consisting of PBS containing 1 mM MgCl_2_, 1% (w/v) SDS (Sigma), 0.1% Triton X-100 (Fisher Lifesciences) and 10 fold protease inhibitor cocktail (Roche Diagnostics, Nutley, NJ). Protein yield and quality was evaluated by gel electrophoresis (Novex electrophoresis system, Invitrogen) followed by silver staining (Invitrogen).

#### Western blot assay

Approximately 30 μg of protein were added to the appropriate volume of 2 × LDS-PAGE sample buffer (Invitrogen) containing 1 mM β-mercaptoethanol and 2 M urea (Sigma Aldrich). The samples were heated at 95°C for 5 mins and electrophoresed on a 10% NuPage bis-Tris gel using the MOPS buffering system followed by transferral to PVDF for 7 min (iBlot, Invitrogen). Filters were blocked in PBS-T containing 5% (w/v) powdered milk (Santa Cruz Biotechnology, Santa Cruz, CA) for 2 hrs. For detection of ApoE the blots were incubated overnight in an HRP conjugated goat anti human ApoE (Academy Biomedical) at 1:500 in block buffer. For detection of GAPDH, blots were probed with a monoclonal antibody at a dilution of 1:1000 (clone 6C5, Chemicon, Temecula, CA). For secondary detection, a rabbit anti-mouse IgG antibody coupled to horse radish peroxidase was used at a dilution of 1:1500 (Sigma). The blots were developed in peroxidase substrate (100 mM Tris pH 8.0 containing 75 μM paracoumaric acid, 500 μM luminol and 0.006% (v/v) hydrogen peroxide, Sigma) for 5 mins prior to exposure to photographic film (Pierce).

### VLDL binding assay

MSCs were transferred to serum free complete culture medium containing Dex or vehicle. After 2 days, the conditioned medium was filtered through a 0.2 μm membrane. One mL of the conditioned medium was added to 50 μg of biotinylated human VLDL solution (Intracel, Fredrick, MD). The mixture was incubated for 4 hr with rotation, and large VLDL aggregates were removed by centrifugation at 15,000 g for 15 min. The mixture was then depleted of biotinylated components by 4 sequential 30 min incubations in wells of a streptavidin coated microtiter plate (Streptawell High Bind, Roche Diagnostics). The supernatant was then subjected to ELISA assay.

### Production and characterization of lipid micelles containing labelled cholesterol ester, cholesterol and MSC derived ApoE

Conditioned serum free medium containing 50–100 ng mL^-1 ^ApoE was incubated in 50 μg mL^-1 ^4,4-difluoro-5-(2-pyrrolyl)-4-bora-3a,4a-diaza-*s*-indacene-3-undecanoate (cholesteryl BODIPY 576/589 C_11_, Invitrogen), and 5 μg mL^-1 ^25-[N-[(7-nitro-2-1,3-benzoxadiazol-4-yl)methyl]amino]-27-norcholesterol (NBD cholesterol). Cholesteryl BODIPY 576/589 C_11 _is a blue cholesteryl ester and NBD cholesterol consists of a cholesterol moiety covalently conjugated to a green fluorophore 460/534. Lipids were allowed to self-assemble at 21°C with slow mixing. In some cases, the micelles were recovered by centrifugation, washed in ice cold PBS and assayed for ApoE content by western blotting. For cell binding studies, Huh-7 hepatocytes (a gift from Srikanta Dash, Tulane Health Sciences Center) were expanded in 6 well plates containing standard media at 37°C with 5% (v/v) CO_2_. Upon initiation of the experiment, 2 mL of micelle-containing serum free medium was added to the cultures and at hourly intervals, cultures were washed in warm PBS, then the medium was replaced by serum free medium without lipid. Cells were visualized by epifluorescent and phase microscopy. Kinetics of the uptake of NBD cholesterol was compared between ApoE containing and vehicle containing control media.

### Statistical Analyses

Measurements were performed in triplicate for each media sample taken and measurements were considered acceptable when the variation was less than 5% of the value. For each condition tested, 3–6 replicate cultures were prepared and assayed. Data were presented as the mean of the measurements from replicate cultures with standard deviations. Representative data are presented from one donor in figures, but multiple donors were assayed yielding the same results.

## Authors' contributions

SZ Conceptualized experimental strategy, carried out experiments, interpreted data, co-wrote manuscript. BSF Carried out experiments, interpreted data. SMH Carried out experiments. MJW Prepared some of the MSCs, carried out experiments. CAG Conceptualized experimental strategy, interpreted data, co-wrote manuscript. DJP Conceptualized experimental strategy, interpreted data, co-wrote manuscript. All authors read and approved the final manuscript.
